# Reducing Behavioral Detection Thresholds per Electrode *via* Synchronous, Spatially-Dependent Intracortical Microstimulation

**DOI:** 10.3389/fnins.2022.876142

**Published:** 2022-06-17

**Authors:** Nicolas G. Kunigk, Morgan E. Urdaneta, Ian G. Malone, Francisco Delgado, Kevin J. Otto

**Affiliations:** ^1^J. Crayton Pruitt Family Department of Biomedical Engineering, University of Florida, Gainesville, FL, United States; ^2^Department of Neuroscience, University of Florida, Gainesville, FL, United States; ^3^Department of Electrical and Computer Engineering, University of Florida, Gainesville, FL, United States; ^4^Department of Materials Science and Engineering, University of Florida, Gainesville, FL, United States; ^5^Department of Neurology, University of Florida, Gainesville, FL, United States; ^6^McKnight Brain Institute, University of Florida, Gainesville, FL, United States

**Keywords:** brain computer interface, intracortical microstimulation (ICMS), detection thresholds, charge density, neuroprostheses

## Abstract

Intracortical microstimulation (ICMS) has shown promise in restoring quality of life to patients suffering from paralysis, specifically when used in the primary somatosensory cortex (S1). However, these benefits can be hampered by long-term degradation of electrode performance due to the brain’s foreign body response. Advances in microfabrication techniques have allowed for the development of neuroprostheses with subcellular electrodes, which are characterized by greater versatility and a less detrimental immune response during chronic use. These probes are hypothesized to enable more selective, higher-resolution stimulation of cortical tissue with long-term implants. However, microstimulation using physiologically relevant charges with these smaller-scale devices can damage electrode sites and reduce the efficacy of the overall device. Studies have shown promise in bypassing this limitation by spreading the stimulation charge between multiple channels in an implanted electrode array, but to our knowledge the usefulness of this strategy in laminar arrays with electrode sites spanning each layer of the cortex remains unexplored. To investigate the efficacy of simultaneous multi-channel ICMS in electrode arrays with stimulation sites spanning cortical depth, we implanted laminar electrode arrays in the primary somatosensory cortex of rats trained in a behavioral avoidance paradigm. By measuring detection thresholds, we were able to quantify improvements in ICMS performance using a simultaneous multi-channel stimulation paradigm. The charge required per site to elicit detection thresholds was halved when stimulating from two adjacent electrode sites, although the overall charge used by the implant was increased. This reduction in threshold charge was more pronounced when stimulating with more than two channels and lessened with greater distance between stimulating channels. Our findings suggest that these improvements are based on the synchronicity and polarity of each stimulus, leading us to conclude that these improvements in stimulation efficiency per electrode are due to charge summation as opposed to a summation of neural responses to stimulation. Additionally, the per-site charge reductions are seen regardless of the cortical depth of each utilized channel. This evocation of physiological detection thresholds with lower stimulation currents per electrode site has implications for the feasibility of stimulation regimes in future advanced neuroprosthetic devices, which could benefit from reducing the charge output per site.

## Introduction

Brain-machine interface (BMI) technology has the capacity to greatly improve quality of life for patients suffering from spinal cord injury, stroke, or amputation. Interfacing directly with the cortex provides high signal quality for stimulating cortical areas in order to produce flashes of light for blind patients (Bak et al., 1990) and touch percepts (Flesher et al., 2016; Salas et al., 2018) for patients with reduced sensory function. Importantly, intracortical microstimulation (ICMS) of the somatosensory cortex (S1) allows for effective sensory feedback in prosthetic BMI systems (O’Doherty et al., 2011, 2019; Flesher et al., 2021), but its highly invasive nature demands careful research into electrode design (Saldanha et al., 2021) and the optimization of parameters (Urdaneta et al., 2021) to improve chronic device integrity and performance.

Advances in fabrication have allowed for the production of smaller implantable electrodes, which produce a less deleterious immune response in nearby tissue (Gunasekera et al., 2015) and can potentially allow for more specific stimulation (Urdaneta et al., 2017). However, smaller probes with lower electrode-site areas may be unable to produce the requisite charges for ICMS without damaging the device or the surrounding tissue (Cogan et al., 2004) due to higher current densities at the electrode sites. To overcome this limitation, multiple electrode sites can deliver stimulation simultaneously, limiting the charge in each site while effectively stimulating the neuronal population (Zaaimi et al., 2013; Kim et al., 2015). Lower stimulation currents can also increase the specificity of stimulation, activating a more homogenous group of neurons in the region around the electrode (Murasugi et al., 1993; Tehovnik et al., 2006). It is important to produce physiologically relevant charges (i.e., for detection) while maintaining current low enough not only to reduce the risk of electrode/tissue damage, but also to ensure high specificity of stimulation.

The simultaneous stimulation of multiple electrode sites has already shown promise in deep brain stimulation (Perlmutter and Mink, 2006; Butson and McIntyre, 2008), in which the technique is used to perform current steering. Moreover, work in the peripheral nervous system has shown promise in the use of synchronous stimulation to increase selectivity and efficiency (Hokanson et al., 2018). To our knowledge, all previous studies involving synchronous ICMS have utilized Utah electrode arrays (UEA) or floating microelectrode arrays (FMA), with simultaneous stimulation performed between topologically arranged electrodes. These experiments found mixed results (Zaaimi et al., 2013; Kim et al., 2015), but their planar electrode arrays had each electrode-site resting in a different cortical column. With novel electrodes, such as the nanoelectronic thread (Luan et al., 2017), Argo (Sahasrabuddhe et al., 2021), and Neuralink (Musk, 2019) devices, multiple low-area electrode sites are present in each shank in the array. The small size of these electrode sites presents a concern for potential stimulation paradigms, which would need to strictly limit the amount of charge used to avoid damage to the electrode. Given the orientation of electrode sites in these probes, simultaneous stimulation from multiple electrodes may present an opportunity for providing effective microstimulation while staying below current density limits. This possibility raises important questions regarding the effectiveness of synchronous stimulation between groups of electrodes closely spaced across cortical depth.

Here, we implanted rats with penetrating arrays containing electrode sites ranging from superficial to deeper layers of the cortex. Using these electrode arrays, we determined the effects of simultaneous multi-electrode stimulation on detection thresholds. Our work shows that simultaneous stimulation of multiple electrodes in an electrode array with stimulation sites spanning cortical depth can result in a reduction in the charge needed per site to elicit a behavioral response. This reduction can be as high as 76% depending on the spacing and number of simultaneously stimulating electrodes. However, increased distance between stimulating electrodes reduces this improvement. Further experiments showed that this effect is independent of cortical depth. Additionally, it appears dependent upon the stimulation waveform polarity of each electrode as well as their synchronicity.

## Results

**FIGURE 1:**
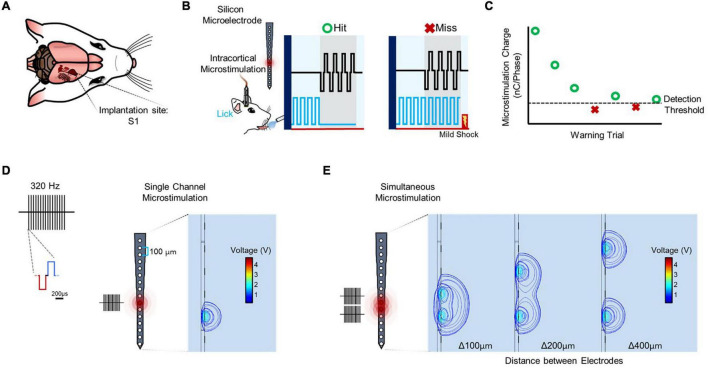
Experimental setup and stimulation paradigm. **(A)** Rat implanted in primary somatosensory cortex (S1). **(B)** Subjects trained in a conditioned avoidance behavioral paradigm in which they are trained to stop licking from a spout with a detector when they feel a stimulus. Every time the subject correctly ceases licking (hit) the amplitude of the stimulus decreases. Conversely, when the subject fails to detect the stimulus (miss) a mild electrical shock is released at the spout and the amplitude of the stimulus increases. **(C)** Trials for a particular set of stimulation parameters are continued until three reversals are detected (miss to hit, hit to miss, miss to hit), at which point the average amplitude of the last five trials are averaged and determined to be the detection threshold. **(D)** Stimulation is delivered from one or more sites spaced 100 μm apart on an electrode shank with sites spanning cortical depth. Each stimulus consists of a 320 Hz train of biphasic, charge-balanced waveforms with a pulse width of 200 μs and variable amplitude. **(E)** Stimulation sites are combined by stimulating from multiple channels simultaneously to obtain data on simultaneous microstimulation thresholds.

Following implantation in primary somatosensory cortex (Figure 1A), detection thresholds were obtained using a modified behavioral avoidance paradigm (Figures 1B,C). Microstimulation consisted of a train of biphasic, charge-balanced waveforms applied from one electrode site (Figure 1D) or simultaneously from multiple electrode sites (Figure 1E; see “Materials and Methods” section).

### Improvement in Detection Thresholds With Synchronous Stimulation

**FIGURE 2:**
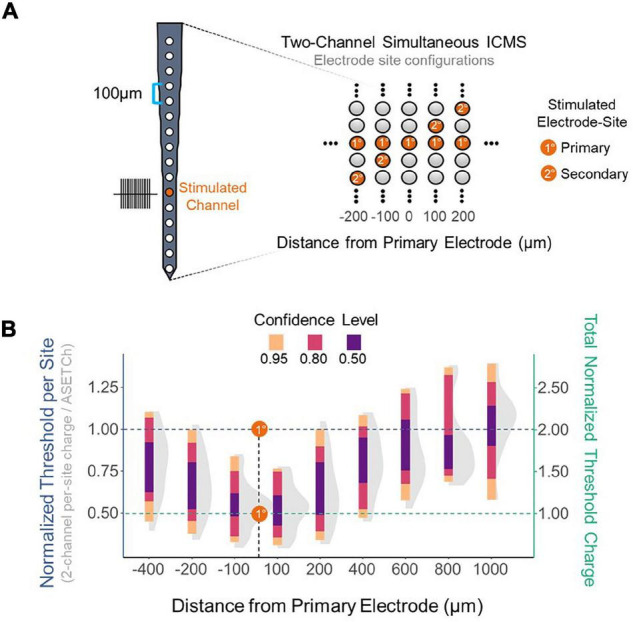
Reduction in per-site threshold charge using simultaneous stimulation. **(A)** Layout of electrode shank and 2-site simultaneous stimulation paradigm. Primary electrode located 1,150 μm from the cortical surface; secondary electrode spaced apart from primary in 100 μm increments. **(B)** Detection thresholds for 2-site synchronous stimulation across different distances from the primary electrode normalized to average single-site threshold. Left Y axis – threshold charge per site divided by average primary electrode threshold charge. Right Y axis – sum of individual electrode threshold charges divided by average primary electrode threshold charge. Dashed lines represent the average single-channel threshold charge when stimulating with the primary electrode-site – normalized to 1.

**FIGURE 3:**
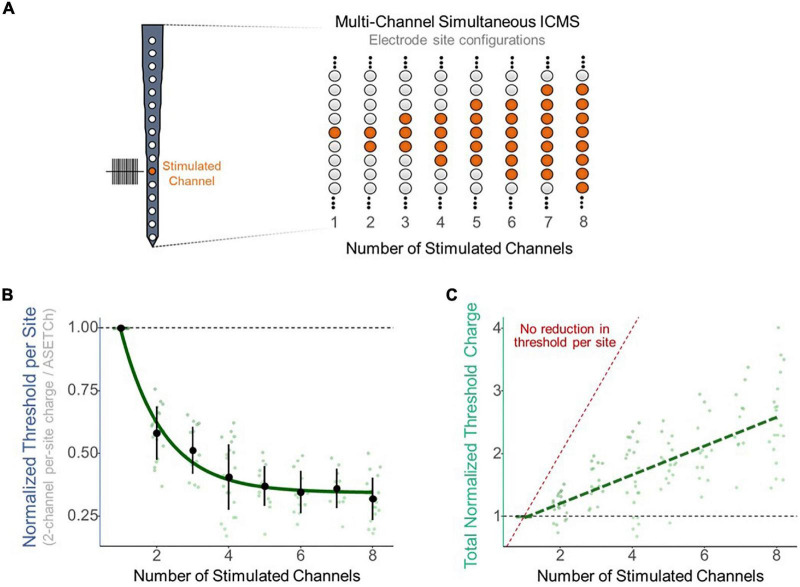
Reduction in per-site threshold charge with multi-site synchronous stimulation. **(A)** Layout of stimulated electrode sites for multi-site experiments. Primary electrode located 1,150 μm from the cortical surface. Increasing number of adjacent secondary electrodes stimulated simultaneously for multi-site stimulation. **(B)** Improvement in per-site threshold charge levels with increasing number of simultaneously stimulated electrode sites. Values were fitted with an exponential decay model (std error = 0.01265). **(C)** Total charge output by the stimulator with increasing number of simultaneously stimulated electrode sites. Values were fitted with a linear regression model (*R*^2^ = 0.641). Red dotted line represents expected total charge values if no reduction in threshold occurred (threshold charge with one channel multiplied by the number of channels stimulated).

To assess the effect of synchronous stimulation on detection thresholds, a stimulation paradigm was implemented which consisted of simultaneously activating a primary electrode at 1,050 μm from the cortical surface and secondary electrodes along the shank (Figure 2A). Simultaneous stimulation thresholds were compared to detection thresholds when stimulating with only the primary electrode. Multisite detection thresholds were measured in per-phase charge per electrode and were normalized each trial by dividing these charge values by the average single-electrode threshold charge (ASETCh) per waveform phase for that animal when stimulating from the primary electrode. All percent values reported reflect a ratio of the obtained multisite threshold divided by the ASETCh, rather than a percent change. For instance, one animal displayed an ASETCh of 4.77 nC/phase. For the same animal, simultaneous stimulation of the primary electrode and a secondary electrode 100 μm deeper resulted in a mean per-site threshold charge of 2.55 nC/phase. In this case, we report that the simultaneous stimulation threshold was 53.5% ASETCh. The average single electrode threshold charge was 5.23 ± 3.28 nC/phase across animals (n = 6). The lowest per-site thresholds obtained occurred when the secondary electrode was closest to the primary electrode and were on average 53.3% ASETCh for the more superficial adjacent site and 55.0% ASETCh for the deeper adjacent site (Figure 2B) (mean = 0.533 × ASETCh ± 0.143 when secondary 100 μm more superficial and mean = 0.550 × ASETCh ± 0.141 when secondary 100 μm deeper). The average multi-channel detection threshold increased as the distance between the primary and secondary electrodes was increased. When the secondary electrode was 200 μm away from the primary site, the average multi-channel threshold was 63.8% ASETCh for a secondary site above the primary and 67.3% ASETCh for a secondary site deeper than the primary (mean = 0.638 × ASETCh ± 0.215 when secondary 200 μm more superficial and mean = 0.673 × ASETCh ± 0.194 when secondary 200 μm deeper). Past 600 μm distance between stimulating electrode sites, the average multi-channel threshold was comparable to single-electrode stimulation (mean = 0.892 × ASETCh ± 0.196 when secondary 600 μm more superficial, mean = 0.931 × ASETCh ± 0.227 when secondary 800 μm more superficial, mean = 1.026 × ASETCh ± 0.231 when secondary 1,000 μm more superficial). To determine whether the improvements seen at small inter-electrode distances could be recreated by increasing the number of synchronously stimulating electrodes, the number of active electrodes was increased up to eight simultaneously stimulating (Figure 3A). In Figure 3B, a clear inverse relationship between the number of stimulated electrodes and the detection threshold level can be seen. The threshold charge per phase decreases rapidly as the number of stimulated electrodes increases from 1 to 4, at which point the improvements in threshold levels compared to single electrode stimulation begin to level off at approximately a third of the ASETCh per phase per electrode-site with an increasing number of electrodes. To further investigate this relationship, an exponential decay model was fitted to these results. The model estimated an asymptote at approximately 34.3% ASETCh (Figure 3B) (Residual standard error: 008.91 on 152 degrees of freedom). Importantly, the total charge injected per phase was not reduced and increased linearly as seen in Figure 3C (Residual standard error: 0.417 on 153 degrees of freedom, *R*^2^ = 0.639).

### Improvement in Threshold Levels Is Independent of Interfacing Depth

**FIGURE 4:**
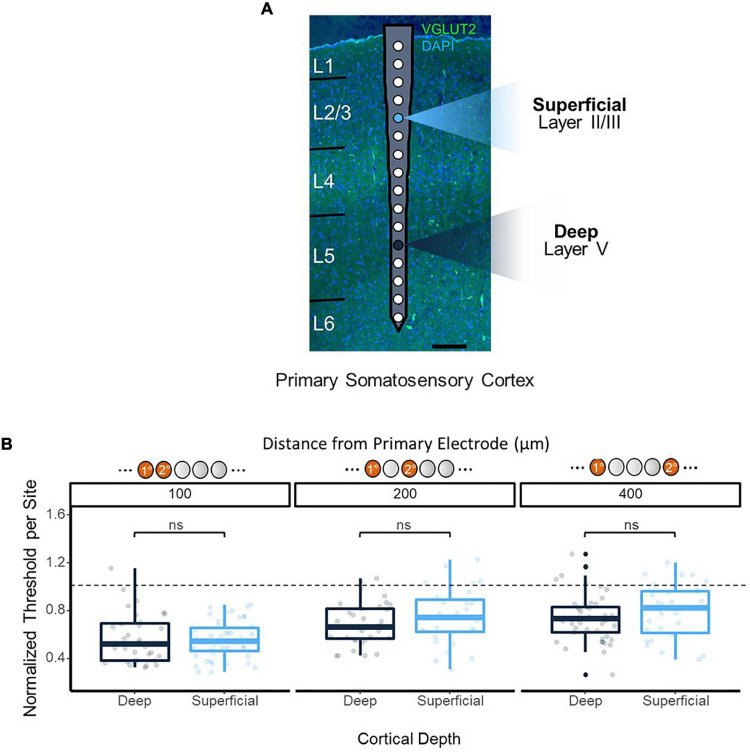
Synchronous stimulation effects over cortical depth. **(A)** Overlay of electrode on histological image to show positioning. Histology performed on coronal section of the implantation site stained with VGLUT2 and DAPI to enable layer identification. Two primary electrode sites were tested: 450 μm from the cortical surface as a shallow site, and 1,150 μm as a deep site (highlighted). **(B)** Consistency of 2-site stimulation effects on threshold over cortical depth. Secondary electrode sites located ±100, 200, and 400 μm from the primary electrode were stimulated, and data from each absolute distance was grouped together. Threshold charges were normalized to the average single-site stimulation threshold charges obtained from the primary electrode site for each depth.

In order to ensure the improvements in threshold levels from synchronous multi-electrode stimulation were not a result of the variations in detection threshold levels across depth shown in previous studies (Koivuniemi and Otto, 2012; Urdaneta et al., 2021), synchronous stimulation thresholds were obtained using reference electrodes at 450 and 1,050 μm from the cortical surface, residing in layers 2/3 and 5 of the cortex, respectively (Figure 4A). No significant difference in thresholds was observed from synchronous stimulation with superficial vs. deep reference electrodes (*p* = 0.59, 100 μm apart; *p* = 0.73, 200 μm apart; *p* = 0.32, 400 μm apart, *t*-test), and both followed the same trend of decreasing improvement in threshold levels as the distance between the reference and test electrodes increased (Figure 4B).

### Improvement in Threshold Levels Is Synchronization-Dependent

**FIGURE 5:**
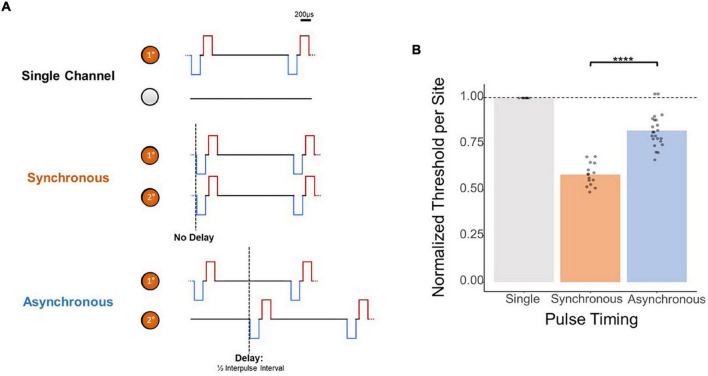
Effect of synchronicity of 2-site simultaneous stimulation on threshold charges. **(A)** Layout of stimulated channels and stimulation paradigm for synchronous and asynchronous experiments. Primary electrode-site located 1,150 μm from the cortical surface; secondary electrode-site located 100 μm deeper than primary site. For synchronous pulses, no delay was present between the onset of stimulus trains for both channels. For asynchronous trials, a delay equal to 1/2 the inter-pulse interval (0.02 ms delay) was added to the secondary channel’s pulses. **(B)** Effect of 2-site simultaneous stimulation synchronicity on per-site threshold charge levels (threshold charges normalized to average single-site primary electrode-site thresholds; ****p ≤ 0.0001).

While these results provide evidence for depth-independent decreases in per-site charge thresholds with multi-electrode simultaneous stimulation, the mechanism behind this improvement is unclear. Next, we attempted to demonstrate that these improvements in thresholds resulted from electrical summation of the waveforms as opposed to solely an increase in the number of neurons simultaneously activated. To this end, we determined the effect of synchronous as opposed to asynchronous stimulation with multiple electrodes by measuring detection thresholds with two synchronized stimulating electrodes as opposed to two stimulating electrodes with a slight timing offset (Figure 5A). The results in Figure 5B from stimulating two adjacent electrodes show that the normalized improvement in thresholds with multi-electrode stimulation is significantly reduced if the pulses are not delivered synchronously (synchronous vs. asynchronous, mean = 0.584 × ASETCh vs. mean = 0.822 × ASETch per site, *p* < 1.1e–6, *t*-test). Compared to the nearly 50% improvement in thresholds seen with synchronous stimulation, reduction in per site thresholds with asynchronous stimulation was under 20%.

### Improvement in Threshold Levels Is Polarity-Dependent

**FIGURE 6:**
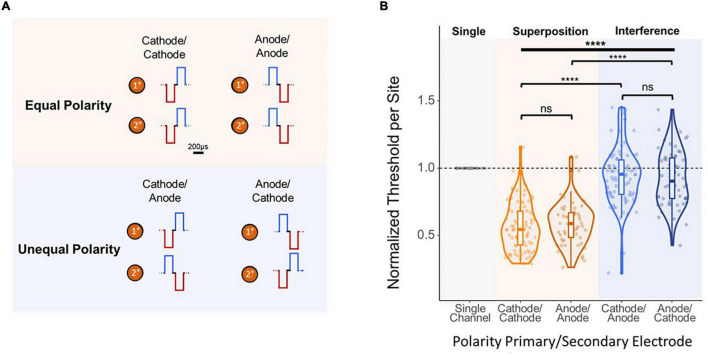
Effect of polarity of 2-site simultaneous stimulation on per-site threshold charges. **(A)** Layout of stimulated channels and stimulation paradigm for equal and opposite polarity experiments. Primary electrode-site located 1,100 μm from the cortical surface; secondary electrode-site located 100 μm deeper than primary site. For equal polarity trials, cathode- or anode-leading waveforms were used in both electrode sites. For opposite polarity trials, each channel used a waveform with a different leading polarity (primary channel cathode-leading with secondary channel anode-leading, and vice-versa). **(B)** Effect of 2-site simultaneous stimulation waveform polarity on per-site threshold charge levels (threshold charges normalized to average single-site primary electrode site thresholds from stimulation with corresponding leading phase polarity; one-way ANOVA (bold line) and *t*-test (brackets) ****p ≤ 0.0001.

Finally, to further analyze the mechanism behind the improvement in threshold levels from simultaneous multi-electrode stimulation, the effect of stimulation waveform polarity was investigated. Thresholds were obtained using stimulation paradigms in which the synchronous pulses were both anode- or cathode-leading (equal), (Figure 6A, top) and in which the pulses had flipped polarities (unequal) (Figure 6A, bottom). Polarity had a significant effect on per-site thresholds [F(3,283) = 93.26, *p* < 2e–16, ANOVA]. Figure 6B shows that both equal cases showed an average per-site threshold charge of under 60% ASETCh (cathode-cathode mean = 0.552 anode-anode mean = 0.591). However, in the unequal polarity case, thresholds were significantly higher, over 90% ASETCh in both cases (cathode-anode mean = 0.947 × ASETCh; anode-cathode mean = 0.926 × ASETCh; cathode-cathode vs. cathode-anode *p* < 1e–16; anode-anode vs. anode-cathode *p* = 3.4e–16, *t*-test). Flipping the order of the phases did not result in any significant improvements in detection threshold levels for either the equal or the unequal polarity stimulation paradigms (cathode-cathode vs. anode-anode: *p* = 0.32; cathode-anode vs. anode-cathode: *p* = 0.55, *t*-test).

## Discussion

Our results demonstrate the utility of synchronous stimulation from multiple electrodes in an intracortical array spanning cortical depth and the effects of various stimulation parameter modifications. This work expands upon previous research investigating the practical utility of simultaneous stimulation of multiple electrodes in a topological array such as the Utah array (Zaaimi et al., 2013; Kim et al., 2015). It also contributes to earlier work investigating the spread of ICMS stimulation current (Stoney et al., 1968) and its relationship with neuronal activation in the cortex (Histed et al., 2009; Kumaravelu et al., 2022).

Advances in microfabrication have enabled the development of novel stimulation devices which utilize smaller probes with a high density of low-area electrode sites that are often oriented along cortical depth (Liu et al., 2015; Xie et al., 2015; Fu et al., 2016; Luan et al., 2017; Musk, 2019). Such probes are desirable for their small size and flexibility, and therefore higher chronic biocompatibility (Gunasekera et al., 2015; Liu et al., 2015; Xie et al., 2015; Fu et al., 2016; Zhou et al., 2017) but may be limited by the damage electrode sites can experience at high current densities, which results in decreased electrode performance (Cogan et al., 2004). The determination from our findings that multiple electrodes in a laminar configuration can be stimulated simultaneously to effectively lower the detection threshold current per site has important implications for these novel devices. Electrochemical research using this type of simultaneous ICMS regime can look into potential redox reaction-induced tissue damage caused by exerting a certain amount of charge from a single electrode-site and compare it to multisite simultaneous stimulation. Future studies could also investigate the effects of different electrode materials (as in Ganji et al., 2017) during simultaneous stimulation. With simultaneous stimulation of multiple channels decreasing detection thresholds by as much as 50% with two adjacent electrode sites, the amount of charge pushed through each site can be reduced and the chronic performance of the electrode improved. This result is in line with previous research (Zaaimi et al., 2013), which showed that increasing the number of closely-spaced stimulating electrodes in a Utah probe increased non-human primate sensitivity to stimulation.

Our experiments with modifications to the synchronicity and polarity of simultaneously delivered ICMS from two channels suggest that this effect is due to the summation of charges from individual electrode sites. With synchronous waveforms (Figure 5A, middle), each phase can exhibit constructive interference with the phase of the other electrode’s stimulus waveform, potentially increasing the effectiveness of stimulation around the areas of the electrode sites. In contrast, simultaneous but asynchronous stimulation (Figure 5A, bottom) would not benefit from this interference due to the temporal offset between electrode sites. In the polarity experiments, the equal polarity case likely experienced the same constructive interference seen with synchronous stimulation waveforms. Alternatively, stimulating with opposing polarity waveforms possibly resulted in destructive interference of the charge injection, producing normalized thresholds barely below the level of single-electrode stimulation. These two modifications imply that the reduction of threshold charge via simultaneous multi-electrode stimulation reported throughout this study is the result of charge summation, as opposed to a summation of separate neural responses to stimulation causing lower threshold charges. Future experiments can investigate the effects of shorter time delays between stimulation pulses as well as the interactions between temporal offsets and differing polarities in multisite ICMS.

As the advancement of BMI technology continues producing smaller probes with more electrode sites, the relevance of improving stimulation paradigms becomes more apparent. With a greater number of channels able to interface across cortical areas and depths, these devices present a unique opportunity to explore multisite stimulation paradigms with increased specificity. Using high-density probes, current steering techniques (Butson and McIntyre, 2008), and dynamic stimulation paradigms (Beauchamp et al., 2020) could become more commonplace in general ICMS paradigms. The use of current steering for deep brain stimulation has been studied as a way to directly affect the volume of tissue activated as well as the shape of the electric field produced during stimulation (Butson and McIntyre, 2008; Hui et al., 2020). Such fine-tuning could be of great use to ICMS paradigms and allow for more selective activation of cortical neurons. Indeed, Beauchamp et al. (2020) successfully utilized current steering in the visual cortex of humans to implement a dynamic stimulation paradigm which allowed patients to identify the shapes of letters and other symbols as they were procedurally stimulated through an electrode array. In this case, simultaneous synchronized stimulation was utilized to increase the capabilities of ICMS, and future paradigms could utilize the greater potential for specificity of high-density probes to further explore the potential of synchronized stimulation methods for microstimulation in S1. These techniques could be applied beyond the somatosensory cortex, increasing the potential of ICMS to restore function to patients via precise multisite stimulation performed in the auditory and visual cortices. The potential of such paradigms combined with high-density probes may also be of importance to ICMS techniques performed in locations within the brain which will likely require highly fine-tuned, location-specific stimulation, such as areas related to memory (Histed and Miller, 2006). These potential paradigms, however, would need to work around the vital limitation of charge-density in each electrode-site. The techniques discussed in this study could benefit future paradigms in advanced devices by enabling effective microstimulation while staying within the charge density limitations of smaller electrode-site areas used in other probes.

While a detection study utilizing multisite stimulation has already been performed in S1 using UEAs and FMAs (Kim et al., 2015), the results from synchronous stimulation with this device differed from those presented here. This discrepancy may arise from the orientation of electrode sites in each array. These topologically-oriented arrays are comprised of multiple shanks of equal length with electrode sites at their tips, placing each stimulating site at roughly the same cortical depth. In contrast, the electrode array used here contains electrode sites arranged across cortical depth, from more superficial at the base of the probe to deeper at the tip. Stimulating with a UEA/FMAs, the authors found no significant difference in detection from single vs. paired electrode stimulation with electrode sites spaced 400 μm apart at the same cortical depth, while our data shows as much as a 25% reduction in thresholds for electrode-site pairs spaced 400 μm apart on the superficial-deep axis. This improvement continued up to a 50% reduction in per-site threshold charge levels at 100 μm apart, which was the smallest spacing possible in the device used here. This difference is likely explained by the orientation of the electrode-site pairs within a cortical column (Mountcastle, 1957), and thus, it may be that only trans-depth arrangements produce such an improvement. It is also important to consider that the authors in Kim et al. (2015) compared the thresholds of multi-electrode stimulation to the threshold of single-electrode stimulation with the most sensitive electrode. Comparing two-electrode thresholds to the mean single-electrode threshold without singling out the lowest-threshold electrode may have yielded a similar result. Future studies could utilize computational modeling to estimate whether the trend continues for smaller distances, both with constant cortical depth and along the superficial-deep axis.

Although the implications of these results may show promise for future BMI technology, further research is needed to determine the extent of their relevance to devices eventually used in a clinical setting. For instance, although our results suggest a potential for decreased current per-site using simultaneous multi-electrode stimulation techniques, it is important to note that this decrease in charge does not translate to a decrease in total charge used. In fact, in nearly all cases, synchronous stimulation resulted in an increase in the charge used by the implant, meaning multisite stimulation would generally decrease battery life for implanted electrode arrays more quickly than standard paradigms. The stimulation parameters not modulated in this study (e.g., pulse train frequency) may also provide useful subjects of investigation in future multisite ICMS studies. Additionally, the behavioral paradigm utilized in these experiments tested only detection in order to determine the feasibility and general usefulness of simultaneous stimulation techniques. However, detection is merely the starting point for clinically relevant ICMS paradigms, and simultaneous multi electrode stimulation may well affect the quality of ICMS-evoked percepts. Future studies could determine qualitative changes in percepts, if any, caused by multi electrode stimulation as opposed to standard single-electrode paradigms. Investigation into these changes could lead to as-yet unknown advantages to simultaneous multi electrode stimulation, adding another potential modulation for more naturalistic, higher-quality percepts.

## Materials and Methods

### Device Implantation Procedure

All animal experiments were performed under the approval and guidance of the Institutional Animal Care and Use Committee (IACUC) of the University of Florida (Gainesville, FL, United States). All surgeries were performed by the same surgeon using aseptic techniques. Prior to implantation, the silicon microelectrode device was sterilized with ethylene oxide. This device had 16 iridium oxide electrode sites (703 um^2^) arranged along the superficial-deep axis of the animal such that they spanned all layers of the cortex (A1 × 16–3 mm-100–703-HZ16, NeuroNexus, Ann Arbor, MI, United States). Six male Sprague-Dawley rats (450–650 g, Charles River, Chicago, IL, United States) were initially induced with 5% isoflurane (Zoetis, Parsippany, NJ, United States) in oxygen at 1.5–2 L/min. The isoflurane was reduced after 5 min and sustained at 1.5–3% throughout the surgery. Meloxicam (1–2 mg/kg, SQ, Loxicom, Norbrook Laboratories, Newry, Northern Ireland) was administered subcutaneously. A 1 mm^2^ cranial window was created over the right forepaw region of the primary somatosensory cortex [0.5 mm anterior to bregma, 3.5 mm lateral to midline (Paxinos and Watson, 2006)] using a microdrill. Four burr holes were drilled to secure titanium bone screws (United Titanium, Wooster, OH, United States). Following creation of a dural slit, an automated micro-insertion system (PiLine M663, Physik Instrumente, Karlsruhe, Germany) was used to insert the microelectrode device 1,600 μm from the cortical surface at 100 mm/s. Correct implantation depth was verified by ensuring complete insertion of the most superficial electrode as well as through electrophysiological and histological assessments of implantation depth described previously (Urdaneta et al., 2022). After implantation, the craniotomy site was filled with silicon elastomer (Kwik-Sil, WPI, Sarasota, FL, United States) followed by layers of UV-cured dental composite (DentalSource, CA, United States) after thickening of the elastomer to secure the electrode and anchor headstage connections.

### Behavioral Paradigm

To determine detection thresholds in freely behaving rats, a modified conditioned avoidance behavioral paradigm (Urdaneta et al., 2019) was implemented. Water-deprived rats were placed in an enclosure with a metal spout capable of both detecting contact and producing a small cutaneous shock. The animal’s licking pattern was monitored, and when the animal contacted the spout for more than 25% of a 200 ms sliding window, a trial was started. Trials were organized into five-trial blocks; four safe trials and one randomly designated warning trail. Safe trials contained no stimulus in order to serve as control trials for inconsistent licking patterns, while warning trials entailed an ICMS stimulus presentation. If the animal successfully withdrew from the spout for over 20% of the stimulus presentation phase during a warning trial, the trial was deemed a hit; otherwise, it was considered a miss. In order to determine each animal’s behavioral detection threshold for a particular stimulation paradigm, an adaptive algorithm was used. The amplitude of the presented stimulus varied depending on animal performance; a miss would result in an increase in stimulus amplitude for the following warning trial, while a hit would result in a decrease. This pattern was repeated, with the magnitude of the amplitude change decreasing with each trial, until three reversals (hit followed by a miss, or vice versa) were recorded. At this point, the average stimulation amplitude of the last five trials was calculated and considered to be the detection threshold for that particular stimulation session.

### Intracortical Stimulation Parameters

Each stimulus was delivered to one or more electrode sites on the implanted device *via* an IZ-32 stimulator with an LZ48-200 battery (Tucker-Davis Technologies, Alachua, FL, United States). All microstimulation experiments used charge-balanced and symmetric biphasic waveforms (Figure 1D). The frequency (320 Hz), duration of pulse trains (650 ms), interpulse interval (0.04 ms), and phase duration (0.2 ms) of the stimuli were kept constant. Unless otherwise stated, stimulation waveforms used in this study were cathode-leading. For each experimental session, an electrode site was selected to serve as the primary electrode, while other sites with varying distances from the reference electrode were chosen to serve as the secondary electrode in random order. Stimulation waveforms were identical for primary and secondary electrode sites. For all experiments reported herein, the total charge injected through each electrode site was limited to 20 nC/phase. Constant stimulation parameters were chosen to be consistent with previous work (Urdaneta et al., 2019, 2021; Saldanha et al., 2021). All stimulation experiments were performed using Synapse stimulation software (Tucker-Davis Technologies, Alachua, FL, United States), which provided the capability to stimulate from more than one electrode-site at once.

### Measuring Effect of Multi-Electrode Stimulation Paradigms

For asynchronous multi-electrode stimulation experiments, the software was configured to send a stimulus through the primary channel and then through the secondary channel after a delay of 0.01 ms, resulting in off-phase stimulation waveforms. In variable polarity experiments, the software was configured such that one or both channels would synchronously stimulate using pulse trains consisting of either cathode- or anode-leading waveforms. For opposite polarity trials, one channel would stimulate with cathode-leading waveforms while the other stimulated with anode-leading, and vice versa. The baseline threshold was defined as the average threshold obtained from stimulating only the primary electrode-site with a cathode-leading waveform train with the parameters outlined in the previous section. For polarity experiments (Figure 6), the baseline threshold was defined as the average threshold obtained by stimulating from only the primary electrode site with a stimulation waveform of the same leading phase polarity as used in the primary electrode site.

### Statistical Analyzes

All statistical analyzes were performed in R Statistical Software Version 4.0.0 (R Core Team, 2018). A Levene’s test was used to test homogeneity of variance between groups and a Shapiro-Wilks test was used to assess normality. Analyzes of variance was performed with one-way ANOVA (Figure 6B) and pairwise comparisons were performed with Student’s *t*-test (Figures 4B–6B). For multichannel stimulation trend analysis, data was estimated to an exponential decay model using “SSAsymp” function of R and fitted using the “nls” function of R.

### COMSOL^®^ Finite Element Analysis

Finite element analysis of ICMS was performed in the COMSOL^®^ Multiphysics software package to investigate voltage fields produced by the implanted electrodes. This is an established approach that has been used to model electric current physics during cortical stimulation (Sankar et al., 2014). The NeuroNexus A16 device used in the behavioral experiments described here was modeled within COMSOL^®^ using device geometry and materials that are public; specifically, an approximately triangular polysilicon shank and circular iridium electrode (30 μm diameter). Cortical tissue was modeled as a bulk material surrounding the electrode with layer-dependent conductivities (Goto et al., 2010). Cortical tissue boundaries were extended to provide a semi-infinite boundary condition. A point source of electric current was placed at the center of the electrode at the electrode-tissue interface. Simplifying assumptions were made including: (1) there is no directional influence on conductivity; (2) all materials have a relative permittivity of 1; (3) the electrode is a uniform conductor with a constant potential distribution across the electrode. The COMSOL^®^ AC/DC module was used to solve the relevant physics. Isopotential lines were plotted for current amplitudes used in the behavioral experiments described here to visualize a simplified model of how synchronous ICMS may be operating to produce the effects described here.

## Data Availability Statement

The raw data supporting the conclusions of this article will be made available by the authors, without undue reservation.

## Ethics Statement

The animal study was reviewed and approved by Institutional Animal Care and Use Committee (IACUC), University of Florida, Gainesville, FL, United States.

## Author Contributions

KO sourced the funding. MU performed the surgical implantations. NK and MU collected and analyzed the microstimulation data. IM performed the modeling. NK wrote the manuscript. All authors conceived and designed the study, reviewed and edited the manuscript, and approved the submitted version.

## Conflict of Interest

The authors declare that the research was conducted in the absence of any commercial or financial relationships that could be construed as a potential conflict of interest.

## Publisher’s Note

All claims expressed in this article are solely those of the authors and do not necessarily represent those of their affiliated organizations, or those of the publisher, the editors and the reviewers. Any product that may be evaluated in this article, or claim that may be made by its manufacturer, is not guaranteed or endorsed by the publisher.
